# Integrated CoNi_2_S_4_ Nanosheets/3D Conductive Scaffold as an Efficient Bifunctional Electrode for High-Performance Supercapacitors and Sensors

**DOI:** 10.3390/mi17040408

**Published:** 2026-03-26

**Authors:** Yaqiang Ji, Junfeng Huang, Weibin Yin, Junrui Xiang, Yongquan Liu, Yongjun Huang, Jingsheng Hong, Long Li

**Affiliations:** 1 School of Mechanical Engineering, Dongguan University of Technology, Dongguan 523808, China; jiyaqiang@dgut.edu.cn (Y.J.); yenwb537@163.com (W.Y.); xiangjr7@163.com (J.X.); liuyongquan_1@163.com (Y.L.); 13728041026@163.com (Y.H.); jingsheng0330@163.com (J.H.); 2 School of Microelectronics, Shenzhen University of Information Technology, Shenzhen 518172, China

**Keywords:** CoNi_2_S_4_ nanosheets, 3D conductive scaffold, supercapacitor, non-enzymatic glucose sensor, bifunctional electrode

## Abstract

Bifunctional materials present a promising route to develop advanced devices, yet the dual performance of CoNi_2_S_4_ nanosheets anchored on a porous scaffold is seldom reported. Herein, we propose a rational fabrication strategy to construct a three-dimensional hierarchical electrode via the in-situ growth of densely aligned CoNi_2_S_4_ nanosheets on a conductive fabric scaffold. This integrated porous architecture concurrently offers an ultrahigh specific surface area, efficient mass transport, and rapid electron conduction. As a supercapacitor, the electrode achieves a high areal capacitance of 3198 mF cm^−2^ at 4 mA cm^−2^ and retains 98.1% of its initial capacitance after 1000 cycles at 20 mA cm^−2^. As a non-enzymatic glucose sensor, it exhibits outstanding selectivity (<4.1% interference), high sensitivity (1049 μA mM^−1^ cm^−2^), a wide linear range (1–8 mM), and a low detection limit (1 μM). These results highlight the significant potential of this binder-free, scaffold-supported nanosheet design for advancing integrated energy storage and biosensing systems.

## 1. Introduction

The rapid growth of integrated and multifunctional electronics, including wearable health monitors [1] and self-powered biosensors [2,3], has intensified the demand for electrode materials that can simultaneously enable electrochemical energy storage and electrochemical sensing within a unified platform [4,5,6]. In addition, the development of such bifunctional systems is driven by the growing need for more intelligent, miniaturized, and highly integrated wearable and implantable medical electronics. Contemporary wearable health-monitoring platforms generally require not only sensing components but also additional modules for signal processing, wireless communication, battery supply, and energy management, which highlights the importance of compact system integration [7]. From this perspective, integrating energy storage and sensing into a single electrochemical platform represents an attractive strategy for reducing device footprint and improving functional compatibility between power supply and signal transduction. Recent proof-of-concept studies have shown that micro-supercapacitors can be integrated with flexible sweat-sensing systems on the same substrate [8], while self-powered biosensor concepts further demonstrate the possibility of using bioelectrochemical energy conversion to simplify sensing architectures and move toward energy-autonomous operation [2]. More broadly, recent advances in wearable and implantable biosensors for closed-loop therapeutic systems, as well as fully integrated microneedle-based platforms for diabetes monitoring and treatment, suggest that combining physiological monitoring with functional intervention in compact bioelectronic systems may open new opportunities for intelligent health management [9,10]. Although these two functions target distinct outputs, both fundamentally rely on efficient interfacial charge transfer and effective species transport at the electrode/electrolyte interface [11,12,13]. Accordingly, the challenge is not simply to identify an active material, but to rationally design an electrode architecture that reconciles three tightly coupled requirements, namely long-term stability in aqueous electrolytes, high electrical conductivity, and abundant electrochemically accessible active sites.

Spinel-type cobalt–nickel sulfide (CoNi_2_S_4_) has been extensively explored as a promising candidate owing to its rich redox chemistry associated with multiple oxidation states of Ni and Co, which is beneficial for pseudocapacitive charge storage and electrocatalytic reactions [14,15,16]. In particular, constructing CoNi_2_S_4_ into ultrathin nanosheet arrays can expose more active sites and shorten ion-diffusion pathways, thereby improving reaction kinetics [17]. However, conventional slurry-cast electrodes suffer from inactive binders and imperfect interparticle contacts, which block active surfaces, increase charge-transfer resistance, and often lead to structural degradation during long-term cycling [18,19,20]. Binder-free integrated electrodes based on in-situ growth on conductive scaffolds have thus been pursued to enhance electrical wiring and active-site utilization [21,22,23].

Despite these advances, developing a robust and high-performance bifunctional electrode in alkaline electrolytes remains nontrivial when Cu is used as the current collector [24,25,26]. Cu offers attractive conductivity, mechanical flexibility, and cost advantages, yet it is susceptible to corrosion and interfacial deterioration under alkaline conditions, which compromises both electrochemical stability and signal reliability [27,28,29]. A direct coating of active sulfides on Cu cannot fully address this issue, because the stability of the entire electrode is governed by the vulnerable Cu/electrolyte interface, while thick protective layers may sacrifice conductivity or mass transport [30,31,32,33]. Consequently, a spatially resolved architecture that can isolate Cu from corrosive environments while maintaining continuous electron pathways and open transport channels is highly desirable but remains insufficiently developed [34,35].

Herein, we report a rationally engineered three-layer architecture consisting of a flexible Cu core, a porous Ni interlayer, and an outer CoNi_2_S_4_ nanosheet network. The porous Ni layer serves as a multifunctional interphase that passivates the Cu substrate against alkaline corrosion and simultaneously provides a high-surface-area conductive scaffold for subsequent CoNi_2_S_4_ growth. Meanwhile, the conformal CoNi_2_S_4_ nanosheets offer abundant redox-active sites and maintain efficient electrolyte penetration, enabling high active-material utilization without compromising mass transport. Benefiting from this decoupled yet synergistic design, the integrated electrode exhibits high areal capacitance with robust cycling durability, and it also delivers sensitive and selective non-enzymatic glucose detection. This work thus provides a practical interface-engineering strategy to stabilize Cu-based multifunctional electrodes and to unify energy-storage and sensing functions in alkaline systems.

## 2. Materials and Methods

### 2.1. Materials

All chemical reagents were of analytical grade and used as received without further purification. Copper sulfate pentahydrate, sodium hydroxide, ethylenediaminetetraacetic acid disodium salt, potassium ferrocyanide, potassium sodium tartrate, formaldehyde solution, copper acetate, nickel chloride, cobalt chloride, ammonium chloride, thiourea, glucose, and polyvinyl alcohol were purchased from Aladdin Chemical Reagent Co., Ltd. (Shanghai, China). Xylose, Fructose, ascorbic acid, maltose, and sodium chloride were obtained from Sinopharm Chemical Reagent Co., Ltd. (Shanghai, China). The fabric substrate was purchased by Taobao Co., Ltd. (Hangzhou, China). Deionized (DI) water from a Milli-Q system (Merck KGaA, Darmstadt, Germany) with a resistivity of 18.2 MΩ cm was used to prepare solutions and wash samples.

### 2.2. Fabrication of 3D Cu@porous Ni Fabric Electrodes with CoNi_2_S_4_ Nanosheets

#### 2.2.1. Preparation of 3D Cu@porous Ni Fabric Substrates

The 3D Cu@porous Ni fabric electrodes were fabricated using a femtosecond laser-activated metal deposition method, which combines low-cost ELD and laser processing, as reported in our previous work [36,37]. Initially, a fabric film was impregnated with several drops of 1 M copper acetate (CuAc_2_) aqueous solution and dried. A Yb: KGW femtosecond laser (Pharos-10 W, Light Conversion, Vilnius, Lithuania) with a tunable repetition rate and a center wavelength of 515 nm (second-harmonic generation) was used as the laser source for all laser-activated processes. In this work, the laser fluence, scanning speed, and repetition rate were set to 22 mJ cm^−2^, 10 mm s^−1^, and 10 kHz, respectively, unless otherwise specified. A tic-tac-toe scanning pattern with a line interval of 15 µm was employed to define the electrode structure. Subsequently, a Cu layer was deposited onto the laser-patterned fabric film via ELD. The ELD was conducted by immersing the sample in a plating bath (maintained at 50 °C for 20 min) consisting of a 1:1 mixture of solution A and solution B. Solution A was an aqueous solution containing copper sulfate pentahydrate (14 g L^−1^), sodium hydroxide (NaOH, 14 g L^−1^), potassium sodium tartrate (14 g L^−1^), ethylenediaminetetraacetic acid disodium salt (20 g L^−1^), and potassium ferrocyanide (10 mg L^−1^). Solution B was a formaldehyde aqueous solution (12 mL L^−1^). Following the Cu deposition, a porous Ni layer was electrodeposited onto the Cu-coated fabric substrate using a dynamic hydrogen bubble template method [38,39]. In this process, the Cu substrate served as the cathode, and a Pt plate served as the anode. The porous Ni interlayer was deposited galvanostatically at 2.5 A cm^−2^ for 60 s using a regulated DC power supply. The electrolyte consisted of 0.1 M nickel chloride and 2 M ammonium chloride in deionized water. The mass loading of porous Ni interlayer was determined to be approximately 15 mg cm^−2^.

#### 2.2.2. Electrodeposition of CoNi_2_S_4_ Nanosheets

The CoNi_2_S_4_ nanosheets were grown onto the as-prepared 3D Cu@porous Ni fabric electrodes via electrochemical deposition. The deposition bath (100 mL) contained 7.5 mM nickel chloride, 5 mM cobalt chloride, and 0.75 M thiourea. The electrochemical deposition was carried out in a three-electrode cell using a Cu@porous Ni fabric electrode as the working electrode, a platinum plate as the counter electrode, and Ag/AgCl as the reference electrode. Cyclic voltammetry was performed at a scan rate of 5 mV s^−1^ for 5 cycles with a potential range of −1.2 to 0.2 V vs. Ag/AgCl at room temperature. After deposition, the samples were thoroughly rinsed with ethanol and deionized water, and then dried under vacuum at 50 °C for 12 h. The mass loading of CoNi_2_S_4_ nanosheets was determined to be approximately 0.8 mg cm^−2^.

### 2.3. Assembly of Flexible Solid-State Supercapacitors

A polyvinyl alcohol/potassium hydroxide (PVA/KOH) gel electrolyte was prepared by dissolving 5 g of PVA and 3 g of KOH in 50 mL of deionized water at 90 °C under continuous stirring until a transparent solution was obtained. The fabricated electrodes were immersed in the gel electrolyte for 5 min and then allowed to solidify at room temperature. Finally, a symmetric flexible solid-state supercapacitor was assembled by sandwiching the gel electrolyte between two identical electrodes, followed by encapsulation with polydimethylsiloxane (PDMS).

### 2.4. Characterization

The morphology and microstructure of the synthesized electrodes were characterized by field-emission scanning electron microscopy (FE-SEM, Nova Nano SEM 450, FEI, Hillsboro, OR, USA) operated at 5 kV. Further structural characterization was carried out using high-resolution transmission electron microscopy (HRTEM, Tecnai G2 F20, FEI, Hillsboro, OR, USA), and the corresponding elemental distributions were analyzed by TEM-EDS at an accelerating voltage of 200 kV. The crystal structure was examined by X-ray diffraction (XRD, Rigaku D/Max 2500, Rigaku, Akishima-shi, Tokyo, Japan) using Cu Kα radiation (λ = 1.5418 Å). The surface elemental composition and chemical states were analyzed by X-ray photoelectron spectroscopy (XPS, PHI-1800, ULVAC-PHI, Chigasaki, Kanagawa, Japan) with monochromatic Al Kα radiation, and all binding energies were calibrated using the C 1 s peak at 284.8 eV.

### 2.5. Electrochemical Measurements

All electrochemical measurements were carried out at room temperature using a standard three-electrode system connected to an electrochemical workstation (CHI 660E, Shanghai Chenhua Instrument Co., Ltd., Shanghai, China). In the system, the prepared samples served as the working electrode, a platinum-plate electrode as the counter electrode, and a mercuric/mercuric oxide electrode as the reference electrode. Cyclic voltammetry, galvanostatic charge–discharge, electrochemical impedance spectroscopy, and chronoamperometry tests were performed to evaluate the electrochemical performance.

## 3. Results and Discussion

### 3.1. Morphology and Composition of the 3D Cu@porous Ni/CoNi_2_S_4_ Electrode

**Figure 1:**
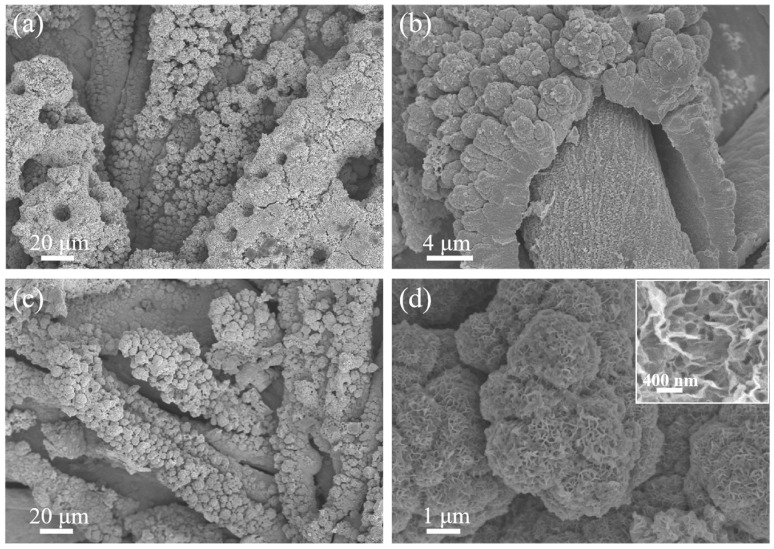
SEM images of (**a**,**b**) the Cu@porous Ni substrate and (**c**,**d**) the Cu@porous Ni/CoNi_2_S_4_ electrode.

Copper-based substrates present a promising platform for flexible energy storage devices due to their inherent conductivity and structural adaptability [40,41,42,43]. However, their susceptibility to corrosion in alkaline electrolytes constitutes a critical limitation for electrochemical applications [44,45,46]. To address this challenge, we developed a stepwise electrodeposition strategy to construct a hierarchical multifunctional electrode architecture. Initially, a porous nickel interlayer was engineered onto the Cu fabric through galvanostatic deposition at 2.5 A cm^−2^ for 60 s. The deposition was performed in an electrolyte containing 0.1 M nickel chloride and 2 M ammonium chloride, where ammonium chloride served dual functions as both a pH buffer and a morphology-directing agent [47]. Under such high-current deposition conditions, porous Ni formation is generally associated with the dynamic hydrogen bubble template mechanism, in which Ni deposition proceeds concurrently with vigorous H_2_ evolution, and the transiently generated hydrogen bubbles act as dynamic templates for pore initiation and growth [48,49]. As a result, the formation of the porous Ni scaffold in the present system can be attributed to the coupled effect of rapid Ni electrodeposition and simultaneous gas evolution, which together promote the development of an open and roughened interlayer morphology. As revealed by SEM (Figure 1a,b), the deposited porous Ni formed a continuous yet highly textured coating composed of densely packed nanoparticles. This transformation converted the originally smooth Cu fibers into a mechanically robust Cu@porous Ni core–shell framework, with the precisely designed interlayer fulfilling dual functions. It simultaneously acted as an effective corrosion barrier to protect the underlying Cu current collector while amplifying the electroactive surface area, thereby creating an optimal three-dimensional conductive scaffold for subsequent active material integration. It should be noted that the apparent gap locally visible in Figure 1b is mainly caused by sample preparation during SEM observation of the rough flexible substrate, rather than actual large-area interfacial delamination.

Building upon this optimized substrate, pseudocapacitive CoNi_2_S_4_ nanosheets were uniformly grown via electrochemical deposition. The resulting composite structure (Figure 1c,d) exhibited remarkable preservation of the intrinsic porous network of the Cu@ porous Ni scaffold while achieving conformal wrapping of its surface with interconnected CoNi_2_S_4_ nanosheets. The outer CoNi_2_S_4_ component is more accurately described as an interconnected nanosheet coating grown on the porous Ni scaffold, rather than as an independently established porous layer. This structural configuration maintained hierarchical ion and electron transport pathways while introducing abundant redox-active sites. The material adopts a core–shell configuration where a flexible Cu core ensures current collection, a porous Ni middle layer offers corrosion resistance and surface enhancement, and a CoNi_2_S_4_ nanosheet outer layer is responsible for charge storage. This spatially resolved design represents a synergistic approach that effectively decouples the traditionally competing requirements of stability, conductivity, and electrochemical activity. The protective Ni interlayer not only passivates the Cu substrate but also actively contributes to the conductive network, while the conformal CoNi_2_S_4_ coating ensures high active material utilization without compromising mass transport efficiency. To further clarify the formation process of the CoNi_2_S_4_ coating, the corresponding cyclic electrodeposition curves are provided in Figure S1. In the thiourea-containing electrolyte, sulfur-containing species generated during cathodic polarization can react with Ni^2+^ and Co^2+^ near the electrode surface, thereby promoting the nucleation of mixed nickel-cobalt sulfide deposits [50,51]. With repeated potential cycling, these nuclei progressively grow and interconnect, leading to the formation of the nanosheet-like CoNi_2_S_4_ network observed on the Cu@porous Ni scaffold.

**Figure 2:**
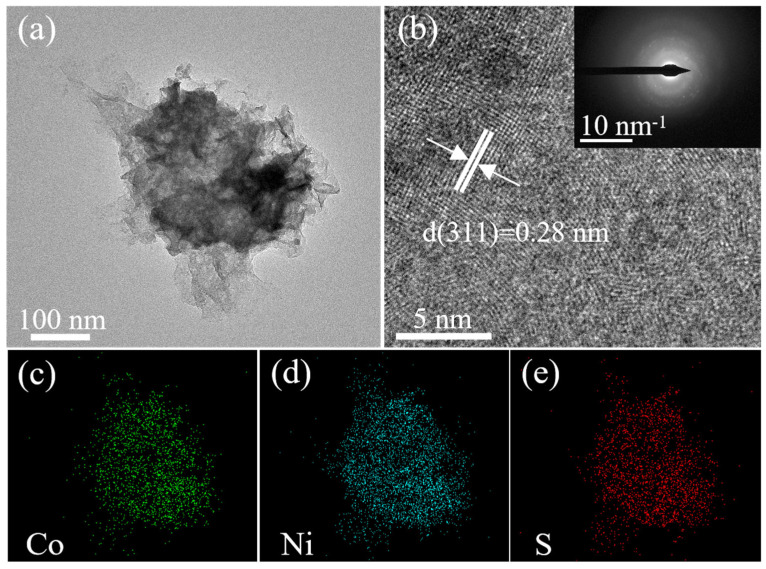
(**a**) TEM images, (**b**) HRTEM, and (**c**–**e**) EDX mapping of CoNi_2_S_4_ nanosheets.

The morphology, crystallinity, and elemental distribution of the synthesized CoNi_2_S_4_ nanosheets were investigated using transmission electron microscopy (TEM). As shown in Figure 2a, the low-magnification TEM image clearly reveals a well-defined sheet-like morphology. Further analysis by high-resolution TEM (HRTEM) in Figure 2b confirms the high crystallinity of the nanosheets. The measured interplanar spacing of 0.28 nm corresponds to the (311) plane of cubic CoNi_2_S_4_. The corresponding fast Fourier transform (FFT) pattern, presented as an inset, exhibits sharp diffraction spots, which further corroborate the single-crystalline nature and excellent crystallographic quality of the product. Moreover, energy-dispersive X-ray (EDX) elemental mapping performed on the nanosheet in Figure 2c–e demonstrates the homogeneous spatial distribution of Co, Ni, and S elements throughout the nanosheet architecture. This uniform elemental dispersion, combined with the observed crystalline structure, is indicative of a phase-pure and homogeneous CoNi_2_S_4_ material, which is crucial for its subsequent electrochemical applications. In addition, semi-quantitative EDS analysis gave elemental contents of Co, Ni, and S of 14 wt.%, 29 wt.%, and 54 wt.%, respectively, which are close to the expected stoichiometric ratio of Co:Ni:S = 1:2:4.

**Figure 3:**
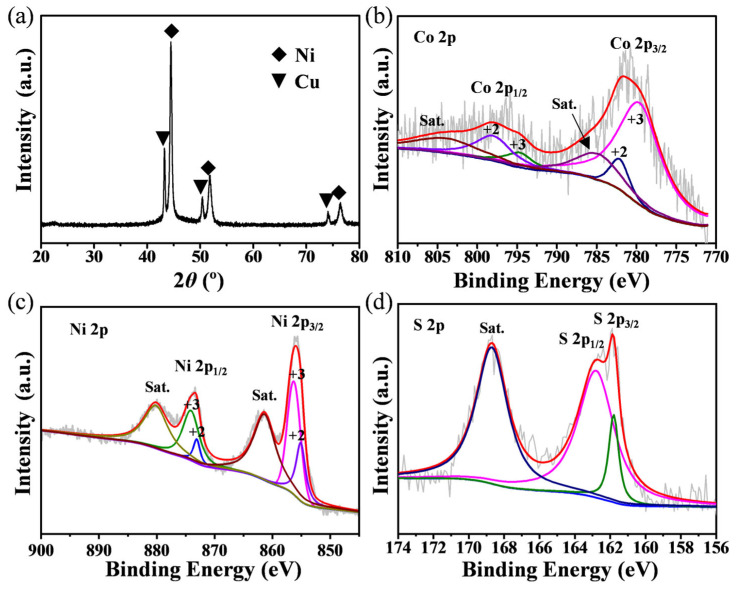
(**a**) XRD spectrum of the Cu@porous Ni/CoNi_2_S_4_ electrode. XPS spectra of (**b**) Co 2p, (**c**) Ni 2p, and (**d**) S 2p.

The composition and chemical states of the hierarchically structured Cu@porous Ni/CoNi_2_S_4_ electrode were systematically examined using X-ray diffraction (XRD) and X-ray photoelectron spectroscopy (XPS). As shown in Figure 3a, the XRD pattern exhibits distinct diffraction peaks corresponding to metallic Cu and Ni, confirming the preservation of the conductive Cu@porous Ni scaffold after the sequential deposition processes. Notably, no distinct diffraction peaks of CoNi_2_S_4_ are observed. This is mainly ascribed to the ultrathin nanosheet nature and/or low loading of the electro-deposited CoNi_2_S_4_, whose weak diffraction signals are readily masked by the intense reflections from the highly crystalline Cu@porous Ni scaffold. Such substrate-dominated XRD patterns are commonly reported for thin, conformal sulfide/oxide coatings on metallic current collectors [52], and the successful formation of CoNi_2_S_4_ is therefore further corroborated by the TEM lattice fringes and XPS results.

To gain deeper insight into the surface chemistry and oxidation states of the constituent elements, the surface-deposited CoNi_2_S_4_ nanosheets were gently scraped from the sample and collected for high-resolution XPS analysis. The Co 2p spectrum (Figure 3b) displays two spin–orbit doublets: the peaks located at binding energies of 782.1 eV and 798.0 eV are assigned to Co^2+^, while those at 779.6 eV and 794.7 eV correspond to Co^3+^, indicating the coexistence of multiple cobalt oxidation states in the deposited sulfide [53]. Similarly, the Ni 2p spectrum (Figure 3c) reveals the presence of both Ni^2+^ (peaks at 855.1 eV and 873.1 eV) and Ni^3+^ (peaks at 856.3 eV and 874.1 eV), suggesting that nickel also exists in mixed valence states, which is beneficial for enhancing redox activity in supercapacitor applications [54]. Furthermore, the S 2p spectrum (Figure 3d) exhibits a peak at 162.8 eV characteristic of metal-sulfur bonds (Co-S and Ni-S), confirming the formation of the sulfide phase. An additional peak at 161.3 eV is attributed to low-coordination S^2−^ species, which often contribute to improved electrochemical reactivity [55]. Collectively, the XPS results corroborate the successful integration of Cu@porous Ni/CoNi_2_S_4_ with a mixed-valence cation environment, while the XRD data reflect the structural dominance of the conductive Cu@porous Ni framework, a configuration that supports both efficient charge transport and rich surface redox chemistry.

### 3.2. Electrochemical Performance of Cu@porous Ni/CoNi_2_S_4_ Electrode for Supercapacitors

**Figure 4:**
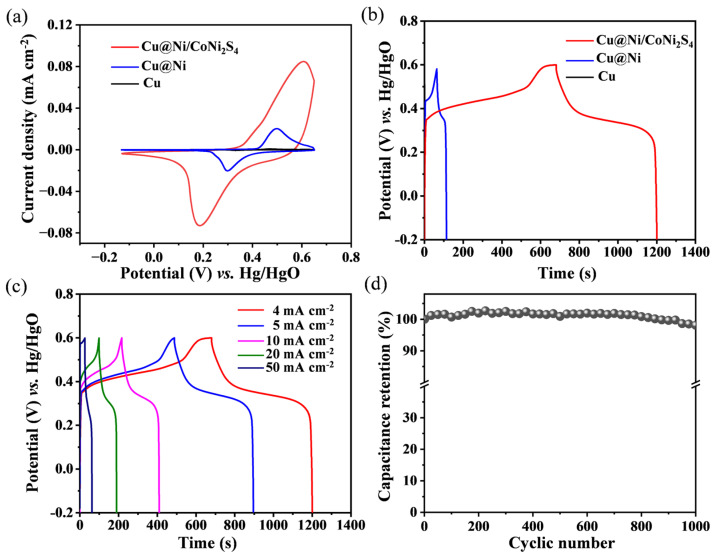
(**a**) CV curves of Cu@porous Ni/CoNi_2_S_4_ electrode, Cu@porous Ni electrode, and Cu electrode. (**b**) GCD curves at 4 mA cm^−2^ current density. (**c**) GCD curves of Cu@porous Ni/CoNi_2_S_4_ electrode at different current densities. (**d**) Cyclic stability test of Cu@porous Ni/CoNi_2_S_4_ electrode at 20 mA cm^−2^ current density.

Benefiting from the hierarchically integrated Cu@porous Ni/CoNi_2_S_4_ nanoarchitecture discussed above, which provides efficient electron/ion transport pathways and abundant electroactive sites, the electrode is expected to deliver high specific capacitance, excellent rate capability, and robust cycling stability. Therefore, its electrochemical energy-storage performance was systematically evaluated in a three-electrode configuration using 1 M KOH electrolyte. Cyclic voltammetry (CV) curves at 10 mV s^−1^ (Figure 4a) revealed distinct redox peaks for both the Cu@porous Ni/CoNi_2_S_4_ and Cu@porous Ni electrodes, indicative of Faradaic pseudocapacitance, whereas the pure Cu electrode showed a featureless curve, confirming its negligible contribution [56,57]. Notably, the significantly larger integrated area under the CV curve for the Cu@porous Ni/CoNi_2_S_4_ composite compared to the Cu@porous Ni substrate unequivocally demonstrates a substantial enhancement in charge storage capacity, directly attributable to the introduction of the highly active CoNi_2_S_4_ nanosheets. This conclusion is corroborated by galvanostatic charge–discharge (GCD) tests. At a current density of 4 mA cm^−2^ (Figure 4b), the Cu@porous Ni/CoNi_2_S_4_ electrode exhibited a markedly prolonged discharge duration over its Cu@porous Ni counterpart, with the Cu electrode showing minimal discharge, confirming the hierarchy in capacitive performance (Cu@porous Ni/CoNi_2_S_4_ >> Cu@porous Ni > Cu).

The GCD profiles of the composite electrode across a range of current densities (4 to 50 mA cm^−2^, Figure 4c) displayed well-defined plateaus and high symmetry. The corresponding areal specific capacitances were calculated as 3198, 2982, 2729, 2528, and 2163 mF cm^−2^, respectively. While the capacitance decreases with increasing current density, which was commonly attributed to diffusion limitations and incomplete redox utilization at high rates, the electrode retained a respectable 68% of its initial capacitance (from 4 to 50 mA cm^−2^), underscoring its good rate capability [58,59]. Most impressively, the Cu@porous Ni/CoNi_2_S_4_ electrode demonstrated exceptional cycling stability, retaining 98.1% of its initial capacitance after 1000 consecutive GCD cycles at 20 mA cm^−2^ (Figure 4d). This outstanding retention, combined with the high specific capacitance and satisfactory rate performance, positions the Cu@porous Ni/CoNi_2_S_4_ architecture as a highly promising electrode material for durable and efficient energy storage devices.

**Figure 5:**
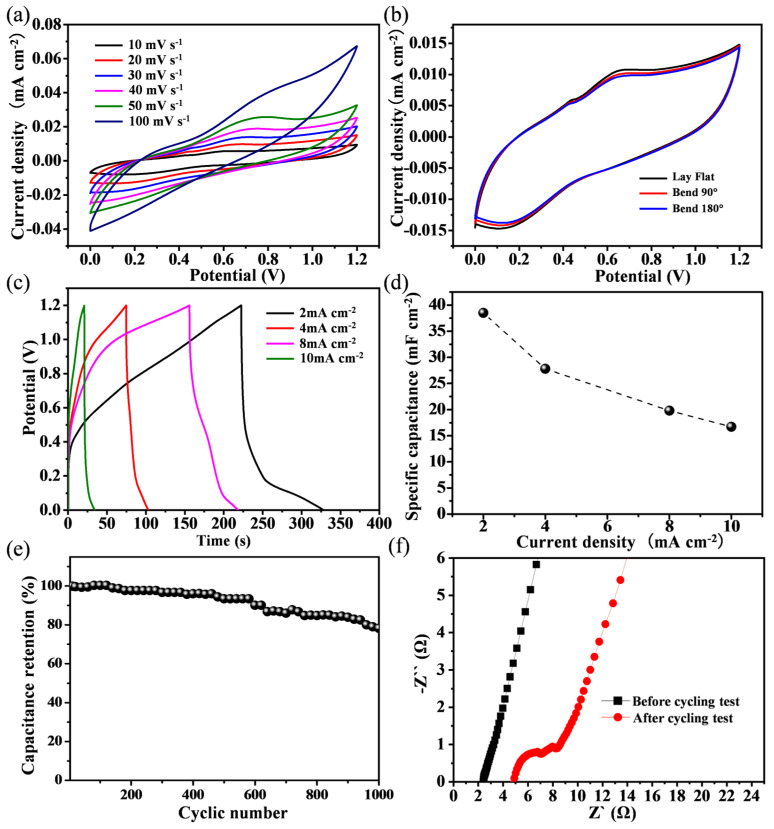
The electrochemical performance of the solid-state supercapacitor. (**a**) CV curves. (**b**) Mechanical stability test. (**c**) GCD curves at different current densities. (**d**) Calculation results of area-specific capacitance. (**e**) Cyclic stability test at 20 mA cm^−2^ current density. (**f**) EIS of the solid-state supercapacitor.

To evaluate the practical viability of the Cu@porous Ni/CoNi_2_S_4_ electrode, a flexible solid-state supercapacitor device was fabricated by sandwiching a PVA/KOH gel electrolyte between two identical electrodes. The electrochemical performance and mechanical robustness of the assembled solid-state supercapacitor were systematically investigated. CV tests conducted at scan rates ranging from 10 to 100 mV s^−1^ (Figure 5a) revealed that the CV curves maintained their shape without significant distortion as the scan rate increased, indicating efficient charge transfer and good rate capability.

The solid-state supercapacitor also showed excellent mechanical flexibility, with nearly identical CV profiles under flat, bent 90°, and bent 180° conditions (Figure 5b), indicating its potential for wearable electronics. GCD measurements at various current densities (2–10 mA cm^−2^, Figure 5c) showed highly symmetric curves, confirming good electrochemical reversibility. The areal specific capacitance calculated from these curves was 83.3, 69.6, 63.3, and 62.5 mF cm^−2^ at 2, 4, 8, and 10 mA cm^−2^, respectively (Figure 5d). The gradual capacitance decrease with increasing current density is attributed to kinetic limitations, where ion diffusion in the electrolyte becomes the rate-limiting step at higher rates. The cycling stability test at 20 mA cm^−2^ (Figure 5e) showed a capacitance retention of 80.1% after 1000 cycles. To gain further insight into the performance degradation, electrochemical impedance spectroscopy (EIS) was performed before and after cycling (Figure 5f). The initial low internal resistance (2.2 Ω) and steep low-frequency slope indicated efficient ion transport at the electrode–electrolyte interface. After cycling, the internal resistance increased to 4.8 Ω, accompanied by a reduced low-frequency slope, suggesting hindered ion diffusion. This increase in resistance, likely caused by gradual dehydration of the unencapsulated gel electrolyte during prolonged operation, is identified as the primary factor for the observed capacitance fade. Overall, the device demonstrates promising flexibility and decent electrochemical performance, while also highlighting that encapsulation to prevent electrolyte dehydration is a critical future step for enhancing long-term stability.

### 3.3. Electrochemical Performance of Cu@porous Ni/CoNi_2_S_4_ Electrode for Glucose Sensors

**Figure 6:**
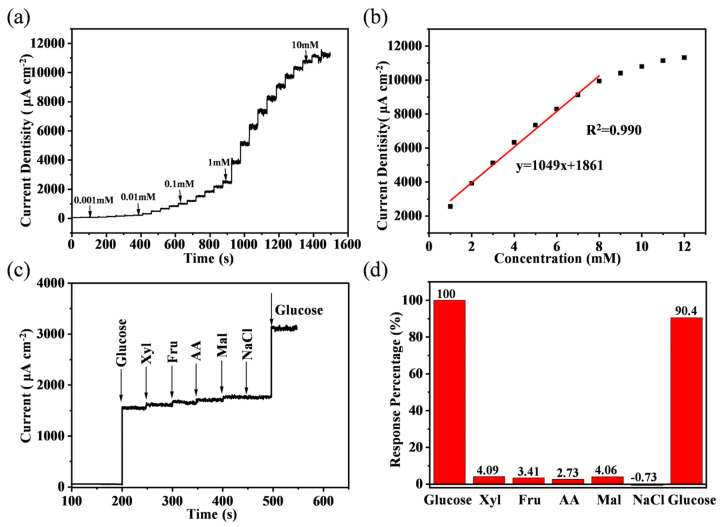
(**a**) Amperometric response curve of the Cu@porous Ni/CoNi_2_S_4_ electrode to successive additions of glucose. (**b**) Corresponding calibration curve and linear fitting. (**c**) Selectivity test. (**d**) The corresponding response percentage.

The glucose-sensing performance of the hierarchical Cu@porous Ni/CoNi_2_S_4_ electrode was evaluated by chronoamperometry in 1 M NaOH. As shown in Figure 6a, the anodic current increased in a rapid and stepwise manner upon successive additions of glucose. This fast response can be attributed to the open porous scaffold [60] and the ultrathin sulfide nanosheet architecture [61], which together facilitate rapid analyte diffusion and efficient charge transport to the electroactive interface. In NaOH solution, the CoNi_2_S_4_ is oxidized to CoS_2*x*_O and NiS_2−*x*_OH [52]. When glucose is added to the solution, Co^3+^ and Ni^4+^ undergo redox reactions with glucose. Co^3+^ is converted to Co^2+^, Ni^4+^ is converted to Ni^3+^, and glucose is oxidized to gluconolactone [62]. The above electrochemical reactions can be explained in the following reaction formula.

When the electrolyte does not contain glucose, the electrochemical reactions are as follows:

CoNi_2_S_4_ + 2OH^−^ ↔ CoS_2*x*_OH + 2NiS_2−*x*_OH + 2e^−^ (1)

CoS_2*x*_OH + OH^−^ ↔ CoS_2*x*_O + H_2_O + e^−^ (2)

After adding glucose, the redox reactions are as follows:

Ni^4+^ + glucose ↔ Ni^3+^ + gluconolactone (3)

Co^3+^ + glucose ↔ Co^2+^ + gluconolactone (4)

The corresponding calibration curve (Figure 6b) exhibited an excellent linear response (R^2^ > 0.990) across a broad glucose concentration range (1–8 mM), from which an ultrahigh sensitivity of 1049 μA mM^−1^ cm^−2^ was determined. A comparison with previously reported non-enzymatic glucose sensors based on transition metal sulfides (Table 1) reveals that the Cu@porous Ni/CoNi_2_S_4_ composite not only offers a wider detection range but also achieves superior sensitivity, underscoring its outstanding performance. Furthermore, a remarkably low limit of detection of ~1 μM was calculated based on a signal-to-noise ratio of 3 (S/N = 3). This exceptional sensitivity and low limit of detection are directly attributable to the synergistic interplay within the Cu@porous Ni/CoNi_2_S_4_ composite: the highly conductive CoNi_2_S_4_ nanosheets provide a robust charge-transfer network, while the surface-anchored Cu@porous Ni nanoparticles offer abundant, highly active sites for the direct electro-oxidation of glucose.

Selectivity is a paramount requirement for reliable sensing in complex biological fluids. The anti-interference properties of the Cu@porous Ni/CoNi_2_S_4_ electrode were systematically investigated by introducing common interferents at physiological concentrations (0.1 mM), including xylose (Xyl), fructose (Fru), ascorbic acid (AA), maltose (Mal), and sodium chloride (NaCl). As depicted in the real-time amperometric response curve (Figure 6c), a sharp and substantial current response was observed upon the first injection of glucose. In contrast, subsequent injections of interfering species induced negligible current variations. Most notably, a second injection of 0.1 mM glucose elicited a pronounced current signal nearly identical to the initial response. This result confirms not only minimal cross-reactivity but also the excellent anti-fouling property and catalytic robustness of the electrode, ensuring that the active sites remain accessible and unpoisoned in a multi-component environment. For quantitative comparison, the responses were normalized to the first glucose signal (Figure 6d). All tested interferents show response levels ≤ 4.1% of glucose, and the electrode retains 90.4% of its original glucose response after the interference sequence, demonstrating robust operational stability and reliable molecular discrimination.

## 4. Conclusions

This work demonstrates the rational design and multifunctional integration of a hierarchical Cu@porous Ni/CoNi_2_S_4_ heterostructure, achieving exceptional performance in both supercapacitor and non-enzymatic glucose sensing applications. The tailored nanoarchitecture, consisting of an electrically conductive Cu@porous Ni scaffold conformally coated with interconnected CoNi_2_S_4_ nanosheets, provides abundant electroactive sites, a large accessible surface area, and efficient pathways for charge and ion transport. For the supercapacitor, the single electrode achieves a high areal capacitance of 3198 mF cm^−2^ at 4 mA cm^−2^ and retains 98.1% of its initial capacitance after 1000 cycles at 20 mA cm^−2^. Furthermore, the assembled flexible solid-state device delivers a specific capacitance of 83.3 mF cm^−2^ and exhibits 80.1% capacitance retention. For glucose sensing, the electrode exhibits an ultrahigh sensitivity of 1049 μA mM^−1^ cm^−2^, a nanomolar detection limit of about 1 μM, and outstanding selectivity with interferent responses below 4.1%. This dual-mode excellence originates from a synergistic design that simultaneously enhances electrical conductivity, promotes rapid mass diffusion, and stabilizes the electrode/electrolyte interface. The work not only establishes a high-performance multifunctional platform but also offers a scalable and versatile blueprint for developing integrated electrochemical systems that bridge advanced sensing and efficient energy storage.

## Figures and Tables

**Table 1:** Comparison of the performances with various non-enzyme glucose sensors.

Electrode	Sensitivity (μA mM^−1^ cm^−2^)	Linear Range (mM)	Limit of Detection (μM)	Ref.
CoNi_2_S_4_@NCF	6.675	0.5–12.5	—	[62]
NiCo_2_S_4_/2D-Carbyne	135	<1	34.5	[63]
Cu_7_S_4_-NiCo_2_S_4_	430	0.002–0.3	0.4	[64]
NiCoS_4_/GCE	858.57	0.005–0.1	1.5	[65]
(Ni-Co)_3_S_4_/GCE	938.4	0.001–5	0.503	[66]
Cu@porous Ni/CoNi_2_S_4_	1049	1–8	1	This work

Note: GCE stands for glassy carbon electrode. NCF stands for nitrogen-doped carbon foam.

## Data Availability

The original contributions presented in this study are included in the article. Further inquiries can be directed to the corresponding authors.

## Supplementary Materials

The following supporting information can be downloaded at: https://www.mdpi.com/article/10.3390/mi17040408/s1. Figure S1: The electrodeposition curve of CoNi_2_S_4_ nanosheets.

## References

[1] Tang W., Sun Q., Wang Z.L. (2023). Self-powered sensing in wearable electronics—A paradigm shift technology. Chem. Rev..

[2] Grattieri M., Minteer S.D. (2018). Self-powered biosensors. ACS Sens..

[3] Reid R.C., Mahbub I. (2020). Wearable self-powered biosensors. Curr. Opin. Electrochem..

[4] Vaghasiya J.V., Mayorga-Martinez C.C., Pumera M. (2022). Telemedicine platform for health assessment remotely by an integrated nanoarchitectonics FePS_3_/rGO and Ti_3_C_2_-based wearable device. npj Flex. Electron..

[5] Duan H., Peng S., He S., Tang S.Y., Goda K., Wang C.H., Li M. (2025). Wearable electrochemical biosensors for advanced healthcare monitoring. Adv. Sci..

[6] Zhang S., Luo S., Xie A. (2025). High-capacity binder-free supercapacitor electrodes obtained from copper foam loaded with large layer spacing NiMn-LDH. J. Energy Storage.

[7] Yokus M.A., Daniele M.A. (2021). Integrated non-invasive biochemical and biophysical sensing systems for health and performance monitoring: A systems perspective. Biosens. Bioelectron..

[8] Lu Y., Jiang K., Chen D., Shen G. (2019). Wearable sweat monitoring system with integrated micro-supercapacitors. Nano Energy.

[9] Zheng Z., Zhu R., Peng I., Xu Z., Jiang Y. (2024). Wearable and implantable biosensors: Mechanisms and applications in closed-loop therapeutic systems. J. Mater. Chem. B.

[10] Li X., Huang X., Mo J., Wang H., Huang Q., Yang C., Zhang T., Chen H.-J., Hang T., Liu F. (2021). A Fully Integrated Closed-Loop System Based on Mesoporous Microneedles-Iontophoresis for Diabetes Treatment. Adv. Sci..

[11] Naikoo G.A., Salim H., Hassan I.U., Awan T., Arshad F., Pedram M.Z., Ahmed W., Qurashi A. (2021). Recent advances in non-enzymatic glucose sensors based on metal and metal oxide nanostructures for diabetes management—A review. Front. Chem..

[12] Rong H., Chen T., Shi R., Zhang Y., Wang Z. (2018). Hierarchical NiCo_2_O_4_@NiCo_2_S_4_ nanocomposite on Ni foam as an electrode for hybrid supercapacitors. ACS Omega.

[13] Nadar N.R., Akkinepally B., Harisha B.S., Ibrahim E.H., Rao H.J., Dash T., Sharma S., Hussain I., Shim J. (2025). Nature-inspired materials as sustainable electrodes for energy storage devices: Recent trends and future aspects. J. Energy Storage.

[14] Liu L., Zuo S., Wang H. (2024). Review of NiCo_2_S_4_-based electrode nanomaterials for supercapacitors. ACS Appl. Nano Mater..

[15] Philip A., Kumar A.R. (2025). Development of a symmetric supercapacitor using a novel NiCo_2_S_4_/CNT composite electrode with ultrahigh energy density. Sci. Rep..

[16] Li D., Gong Y., Pan C. (2016). Facile synthesis of hybrid CNTs/NiCo_2_S_4_ composite for high performance supercapacitors. Sci. Rep..

[17] Lang X., Chu D., Wang Y., Ge D., Chen X. (2022). Defect surface engineering of hollow NiCo_2_S_4_ nanoprisms towards performance-enhanced non-enzymatic glucose oxidation. Biosensors.

[18] Zhang Y., Zhang Y., Zhang Y., Si H., Sun L. (2019). Bimetallic NiCo_2_S_4_ nanoneedles anchored on mesocarbon microbeads as advanced electrodes for asymmetric supercapacitors. Nano-Micro Lett..

[19] Sahoo S., Naik K.K., Rout C.S. (2018). Controlled Electrochemical Growth of Spinel NiCo_2_S_4_ Nanosheets on Nickel Foam for High Performance Supercapacitor Applications. Mater. Today Proc..

[20] Cheng S., Wang X., Du K., Mao Y., Han Y., Li L., Liu X., Wen G. (2023). Hierarchical lotus-seedpod-derived porous activated carbon encapsulated with NiCo_2_S_4_ for a high-performance all-solid-state asymmetric supercapacitor. Molecules.

[21] Ouyang Y., Zhang B., Wang C., Xia X., Lei W., Hao Q. (2021). Bimetallic metal-organic framework derived porous NiCo_2_S_4_ nanosheets arrays as binder-free electrode for hybrid supercapacitor. Appl. Surf. Sci..

[22] Pathak M., Polaki S., Rout C.S. (2022). High performance asymmetric supercapacitors based on Ti_3_C_2_T_x_ MXene and electrodeposited spinel NiCo_2_S_4_ nanostructures. RSC Adv..

[23] Zhang N., Wang M., Quan Y., Li X., Hu X., Yan J., Wang Y., Sun M., Li S. (2025). A review of binder-free electrodes for advanced supercapacitors. J. Ind. Eng. Chem..

[24] Wang X., Xia X., Beka L.G., Liu W., Li X. (2016). In situ growth of urchin-like NiCo_2_S_4_ hexagonal pyramid microstructures on 3D graphene nickel foam for enhanced performance of supercapacitors. RSC Adv..

[25] Ismail M.M., Hong Z.-Y., Arivanandhan M., Yang T.C.-K., Pan G.-T., Huang C.-M. (2021). In situ binder-free and hydrothermal growth of nanostructured NiCo_2_S_4_/Ni electrodes for solid-state hybrid supercapacitors. Energies.

[26] Chen H., Jiang J., Zhang L., Xia D., Zhao Y., Guo D., Qi T., Wan H. (2014). In situ growth of NiCo_2_S_4_ nanotube arrays on Ni foam for supercapacitors: Maximizing utilization efficiency at high mass loading to achieve ultrahigh areal pseudocapacitance. J. Power Sources.

[27] Niu L., Wang Y., Ruan F., Shen C., Shan S., Xu M., Sun Z., Li C., Liu X., Gong Y. (2016). In situ growth of NiCo_2_S_4_@Ni_3_V_2_O_8_ on Ni foam as a binder-free electrode for asymmetric supercapacitors. J. Mater. Chem. A.

[28] Zhang L., Chen W., Zhao Y., Shen L., Cai J., Lu Z., Xiao L., Hou L. (2023). Construction of 3D interconnected NiCo_2_S_4_/bio-carbon coated Ni foam as binder-free electrode for high-performance supercapacitor. Colloids Surf. A Physicochem. Eng. Asp..

[29] Rafique N., Asif A.H., Hirani R.A.K., Wu H., Shi L., Zhang S., Sun H. (2022). Binder free 3D core-shell NiFe layered double hydroxide (LDH) nanosheets (NSs) supported on Cu foam as a highly efficient non-enzymatic glucose sensor. J. Colloid Interface Sci..

[30] Kim D.-Y., Ghodake G., Maile N., Kadam A., Sung Lee D., Fulari V., Shinde S. (2017). Chemical synthesis of hierarchical NiCo_2_S_4_ nanosheets like nanostructure on flexible foil for a high performance supercapacitor. Sci. Rep..

[31] Van Hoa N., Dat P.A., Van Chi N., Quan L.H. (2021). A hierarchical porous aerogel nanocomposite of graphene/NiCo_2_S_4_ as an active electrode material for supercapacitors. J. Sci. Adv. Mater. Devices.

[32] Lin Y., Huang C., Huang C., Deng Y., Zou X., Ma W., Fang G., Ragauskas A.J. (2024). Cellulose regulated lignin/cellulose-based carbon materials with hierarchical porous structure for energy storage. Adv. Compos. Hybrid Mater..

[33] Qi J., Wang D., Zhang Z., Hu R., Sui Y., He Y., Meng Q., Wei F. (2021). Enhanced performance of mesoporous NiCo_2_S_4_ nanosheets fibre-shaped electrode for supercapacitor. Micro Nano Lett..

[34] Tian Y., Yang F., Qiu Z., Jing J., He J., Xu H. (2023). Hierarchical N-Ti_3_C_2_T_X_@ NiCo_2_S_4_ core-shell nanosheets assembled into 3D porous hydrogel as free-standing electrodes for high-performance supercapacitors. J. Energy Storage.

[35] Zhang S., Zhang Q., Ma R., Feng X., Chen F., Wang D., Zhang B., Wang Y., Guo N., Xu M. (2024). Boosting the capacitive performance by constructing O, N co-doped hierarchical porous structure in carbon for supercapacitor. J. Energy Storage.

[36] Ji Y., Zhang Y., Zhu J., Geng P., Halpert J.E., Guo L. (2023). Splashing-assisted femtosecond laser-activated metal deposition for mold-and mask-free fabrication of robust microstructured electrodes for flexible pressure sensors. Small.

[37] Ji Y., Liao Y., Li H., Cai Y., Fan D., Liu Q., Huang S., Zhu R., Wang S., Wang H. (2022). Flexible metal electrodes by femtosecond laser-activated deposition for human-Machine interfaces. ACS Appl. Mater. Interfaces.

[38] Ji Y., Xie J., Wu J., Yang Y., Fu X.-Z., Sun R., Wong C.-P. (2018). Hierarchical nanothorns MnCo_2_O_4_ grown on porous/dense Ni bi-layers coated Cu wire current collectors for high performance flexible solid-state fiber supercapacitors. J. Power Sources.

[39] Ji Y., Xie J., Yang Y., Fu X., Sun R., Wong C. (2020). NiCoP 1D nanothorns grown on 3D hierarchically porous Ni films for high performance hydrogen evolution reaction. Chin. Chem. Lett..

[40] Liu W., Zhai P., Li A., Wei B., Si K., Wei Y., Wang X., Zhu G., Chen Q., Gu X. (2022). Electrochemical CO_2_ reduction to ethylene by ultrathin CuO nanoplate arrays. Nat. Commun..

[41] Pullanchiyodan A., Manjakkal L., Dahiya R. (2021). Metal coated fabric based asymmetric supercapacitor for wearable applications. IEEE Sens. J..

[42] El Halimi M.S., Zanelli A., Soavi F., Chafik T. (2023). Building towards supercapacitors with safer electrolytes and carbon electrodes from natural resources. World.

[43] Fan P., Wang J., Ding W., Xu L. (2023). Core-shell structured carbon nanofiber-based electrodes for high-performance supercapacitors. Molecules.

[44] Nakayama S., Notoya T., Osakai T. (2010). A mechanism for the atmospheric corrosion of copper determined by voltammetry with a strongly alkaline electrolyte. J. Electrochem. Soc..

[45] Chen X., Wu Y., Holze R. (2023). Ag(e)ing and degradation of supercapacitors: Causes, mechanisms, models and countermeasures. Molecules.

[46] Liu S., Li Y., Wang D., Xi S., Xu H., Wang Y., Li X., Zang W., Liu W., Su M. (2024). Alkali cation-induced cathodic corrosion in Cu electrocatalysts. Nat. Commun..

[47] Vainoris M., Tsyntsaru N., Cesiulis H. (2019). Modified electrodeposited cobalt foam coatings as sensors for detection of free chlorine in water. Coatings.

[48] Borges G.G., Medina M., Dos Santos R.M., Dourado A.H., Beluomini M.A., França V.V., Brito J.F.D. (2026). Tailoring Nickel Porous Structure via Dynamic Hydrogen Bubble Template for Efficient Alkaline Hydrogen Evolution. ACS Omega.

[49] Sengupta S., Patra A., Jena S., Das K., Das S. (2018). A Study on the Effect of Electrodeposition Parameters on the Morphology of Porous Nickel Electrodeposits. Metall. Mater. Trans. A.

[50] Irshad A., Munichandraiah N. (2017). Electrodeposited Nickel–Cobalt–Sulfide Catalyst for the Hydrogen Evolution Reaction. ACS Appl. Mater. Interfaces.

[51] Xu S., Su C., Wang T., Ma Y., Hu J., Hu J., Hu N., Su Y., Zhang Y., Yang Z. (2018). One-step electrodeposition of nickel cobalt sulfide nanosheets on Ni nanowire film for hybrid supercapacitor. Electrochim. Acta.

[52] Wang T., Zhao B., Jiang H., Yang H.-P., Zhang K., Yuen M.M., Fu X.-Z., Sun R., Wong C.-P. (2015). Electro-deposition of CoNi_2_S_4_ flower-like nanosheets on 3D hierarchically porous nickel skeletons with high electrochemical capacitive performance. J. Mater. Chem. A.

[53] Sivanantham A., Ganesan P., Shanmugam S. (2016). Hierarchical NiCo_2_S_4_ nanowire arrays supported on Ni foam: An efficient and durable bifunctional electrocatalyst for oxygen and hydrogen evolution reactions. Adv. Funct. Mater..

[54] Chen W., Xia C., Alshareef H.N. (2014). One-step electrodeposited nickel cobalt sulfide nanosheet arrays for high-performance asymmetric supercapacitors. ACS Nano.

[55] Chang P., Mei H., Tan Y., Zhao Y., Huang W., Cheng L. (2020). A 3D-printed stretchable structural supercapacitor with active stretchability/flexibility and remarkable volumetric capacitance. J. Mater. Chem. A.

[56] Shinde S., Ramesh S., Bathula C., Ghodake G., Kim D.-Y., Jagadale A., Kadam A., Waghmode D., Sreekanth T., Kim H.S. (2019). Novel approach to synthesize NiCo_2_S_4_ composite for high-performance supercapacitor application with different molar ratio of Ni and Co. Sci. Rep..

[57] Wang J., Hu L., Zhou X., Zhang S., Qiao Q., Xu L., Tang S. (2021). Three-dimensional porous network electrodes with Cu(OH)_2_ nanosheet/Ni_3_S_2_ nanowire 2D/1D heterostructures for remarkably cycle-stable supercapacitors. ACS Omega.

[58] Han Y., Sun S., Cui W., Deng J. (2020). Multidimensional structure of CoNi_2_S_4_ materials: Structural regulation promoted electrochemical performance in a supercapacitor. RSC Adv..

[59] Zhou L., He Y., Jia C., Pavlinek V., Saha P., Cheng Q. (2017). Construction of hierarchical CuO/Cu_2_O@NiCo_2_S_4_ nanowire arrays on copper foam for high performance supercapacitor electrodes. Nanomaterials.

[60] Chen Z., Zhao M., Lv X., Zhou K., Jiang X., Ren X., Mei X. (2018). Fast ion transport through ultrathin shells of metal sulfide hollow nanocolloids used for high-performance energy storage. Sci. Rep..

[61] Tian Y., Ma Y., Sun R., Zhang W., Liu H., Liu H., Liao L. (2023). Enhanced electrochemical performance of metallic CoS-based supercapacitor by cathodic exfoliation. Nanomaterials.

[62] Dong M., Hu H., Ding S., Wang C., Li L. (2021). Flexible non-enzymatic glucose biosensor based on CoNi_2_S_4_ nanosheets grown on nitrogen-doped carbon foam substrate. J. Alloys Compd..

[63] Dhandapani P., Subbiah Petchimuthuraju A.K., Prasad Rajendra S., AlSalhi M.S., Angaiah S. (2024). Construction of hierarchical NiCo_2_S_4_/2D-Carbyne nanohybrid onto nickel foam for high performance supercapacitor and non-enzymatic electrochemical glucose sensor applications. ChemPhysChem.

[64] Chu T., Zhang C., Huang R., Zhang W., Deng D., Yan X., Zhang Q., Luo L. (2024). NiCo_2_S_4_ nanosheets supported on Cu_7_S_4_ microcubes for the electrochemical detection of glucose in human serum. ACS Appl. Nano Mater..

[65] Chen D., Wang H., Yang M. (2017). A novel ball-in-ball hollow NiCo_2_S_4_ sphere based sensitive and selective nonenzymatic glucose sensor. Anal. Methods.

[66] Meng A., Yuan X., Li Z., Zhao K., Sheng L., Li Q. (2019). Direct growth of 3D porous (Ni-Co)_3_S_4_ nanosheets arrays on rGO-PEDOT hybrid film for high performance non-enzymatic glucose sensing. Sens. Actuators B-Chem..

