# Supplementary material for: Integrated CoNi2S4 Nanosheets/3D Conductive Scaffold as an Efficient Bifunctional Electrode for High-Performance Supercapacitors and Sensors
Source: Micromachines (Basel). 2026 Mar 26;17(4):408. doi: 10.3390/mi17040408 (PMC13117840; doi:10.3390/mi17040408)
Supplement: Supplementary file 1 [file micromachines-17-00408-s001.zip › micromachines-4178357-supplementary.pdf]

# Integrated $CoNi_2S_4$ Nanosheets/3D Conductive Scaffold as an Efficient Bifunctional Electrode for High-Performance Supercapacitors and Sensors

**Yaqiang Ji [1], Junfeng Huang [1,*], Weibin Yin [1], Junrui Xiang [1], Yongquan Liu [1], Yongjun Huang [1], Jingsheng Hong [1] and Long Li [2,*]**

[1] School of Mechanical Engineering, Dongguan University of Technology, Dongguan 523808, China; jiyaqiang@dgut.edu.cn (Y.J.); yenwb537@163.com (W.Y.); xiangjr7@163.com (J.X.); liuyongquan_1@163.com (Y.L.); 13728041026@163.com (Y.H.); jingsheng0330@163.com (J.H.)

[2] School of Microelectronics, Shenzhen University of Information Technology, Shenzhen 518172, China

* Correspondence: 2022146@dgut.edu.cn(J.H.); lilong@sziit.edu.cn(L.L.)

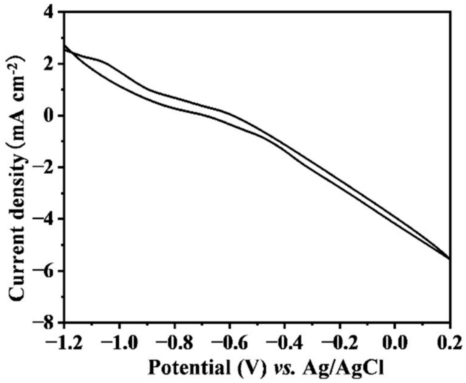


**Figure S1.** The electrodeposition curve of $CoNi_2S_4$ nanosheets.
